# Derivation and validation of the clinical prediction model for COVID-19

**DOI:** 10.1007/s11739-020-02480-3

**Published:** 2020-09-15

**Authors:** Fabrizio Foieni, Girolamo Sala, Jason Giuseppe Mognarelli, Giulia Suigo, Davide Zampini, Matteo Pistoia, Mariella Ciola, Tommaso Ciampani, Carolina Ultori, Paolo Ghiringhelli

**Affiliations:** 1 Internal Medicine, Busto Arsizio Hospital, ASST Valle Olona, Busto Hospital, Varese, Lombardy, Italy; 2 Vascular Surgery, ASST Valle Olona, Busto Hospital, Varese, Lombardy, Italy; 3 Pneumology, ASST Valle Olona, Busto Hospital, Varese, Lombardy, Italy; 4 School of Vascular Surgery, Università degli Studi di Milano, Milan, Italy

**Keywords:** Covid-19, Critical illness, Derivation score, Validation score, Score, Predictive-markers, Sars-CoV2

## Abstract

The epidemic phase of Coronavirus disease 2019 (COVID-19) made the Worldwide health system struggle against a severe interstitial pneumonia requiring high-intensity care settings for respiratory failure. A rationalisation of resources and a specific treatment path were necessary. The study suggests a predictive model drawing on clinical data gathered by 119 consecutive patients with laboratory-confirmed COVID-19 admitted in Busto Arsizio hospital. We derived a score that identifies the risk of clinical evolution and in-hospital mortality clustering patients into four groups. The study outcomes have been compared across the derivation and validation samples. The prediction rule is based on eight simple patient characteristics that were independently associated with study outcomes. It is able to stratify COVID-19 patients into four severity classes, with in-hospital mortality rates of 0% in group 1, 6–12.5% in group 2, 7–20% in group 3 and 60–86% in group 4 across the derivation and validation sample. The prediction model derived in this study identifies COVID-19 patients with low risk of in-hospital mortality and ICU admission. The prediction model that the study presents identifies COVID-19 patients with low risk of in-hospital mortality and admission to ICU. Moreover, it establishes an intermediate portion of patients that should be treated accurately in order to avoid an unfavourable clinical evolution. A further validation of the model is important before its implementation as a decision-making tool to guide the initial management of patients.

## Introduction

Coronavirus disease 2019 (COVID-19) is the third coronavirus infection of the past two decades, after severe acute respiratory syndrome (SARS) and Middle East respiratory syndrome (MERS) [1, 2]. As the COVID-19 pandemic spreads worldwide, intensive care unit (ICU) practitioners, hospital administrators, governments, policy makers, and researchers must prepare for a surge in critically ill patients. The number of people with COVID-19 worldwide crossed the one million mark on April 2, 2020; the case fatality rate across 204 countries and territories was 5·2% [3]. In a large report, 49% of all 2087 critically ill patients with COVID-19 in China died [4, 5]. Small, single-ICU studies found mortality rates of 62% (in Wuhan, China) and 52% (in Washington, DC, USA), but these figures had not accounted for many who were still in the ICU. Although 97% of patients on invasive mechanical ventilation died in a multicenter study conducted early in the Wuhan outbreak, mortality is affected by local practices, and larger studies are awaited [6]. Risk stratification in the acute phase of COVID-19 is of paramount importance, because it may help guide decision making in terms of the intensity of the initial treatment during an acute phase and duration of treatment. Prediction of the trajectory of illness from symptom onset is difficult, and prognostic tools and biomarkers are urgently needed. We, therefore, sought to develop a practical clinical prediction rule for patients with covid-19 that identify patients at risk of in-hospital mortality and admission in ICU and that relies only on readily available clinical parameters and ordinary laboratory tests.

## Methods

This study was conducted urgently during the outbreak of COVID-19 in compliance with the declaration of Helsinki, good clinical practice guidelines, and local regulatory requirements (Lombardy, Italy). Written Institutional informed consent about privacy and personal data management was acquired at the presentation to hospital emergency room.

### Patient identification and eligibility

We obtained the medical records and compiled data for 119 consecutive hospitalized patients with laboratory-confirmed COVID-19 from the Busto Arsizio Hospital (Varese, Italy) from 15 March to 30 April 2020. A confirmed case of COVID-19 was defined as a positive result on real-time reverse-transcriptase–polymerase-chain-reaction (RT-PCR) assay of nasal-pharyngeal swab specimens [7]. Alternative respiratory specimen collection in the intubated patient included tracheal aspirates and bronchoscopy alveolar lavage.

### Study definitions

**Fig. 1:**
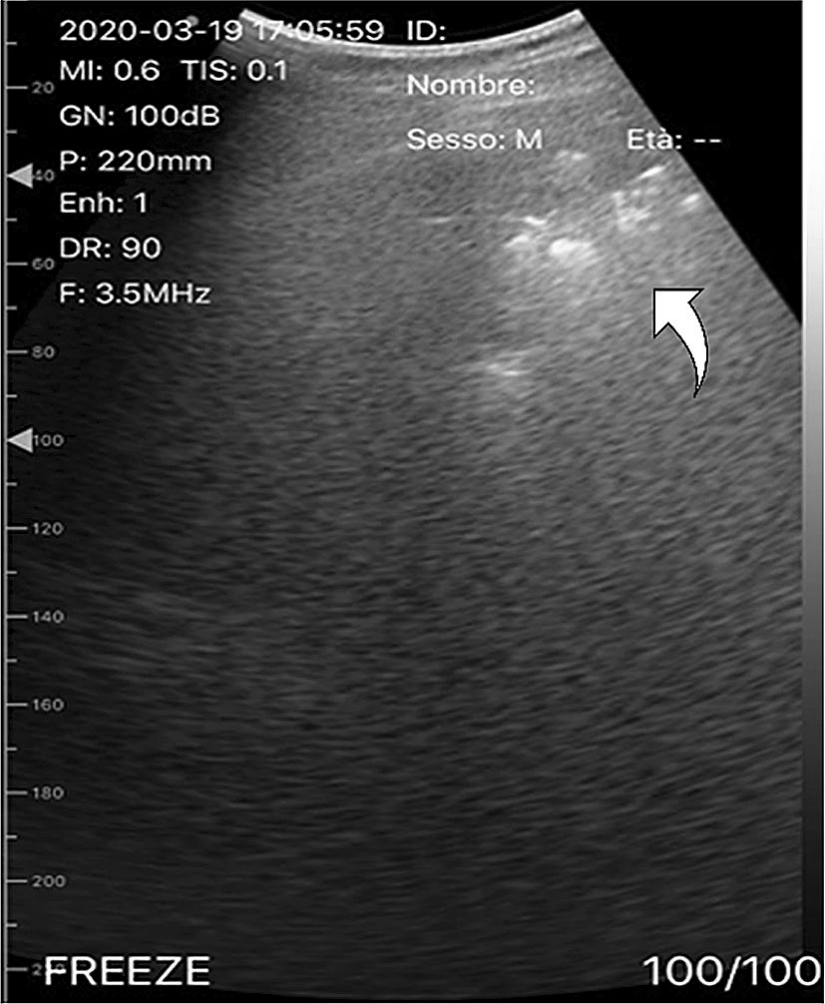
Pulmonary ultrasound pattern “wet” (the arrow indicates a consolidation with subpleural consolidations)

Fever was defined as an axillary temperature of 37.5 °C or higher. The arterial oxygen saturation in room air (SpO2) was measured on arrival of the patient in Emergency Room with a CE certified Pulse Oximeter Fingertip and at the same time a blood gas analysis was also performed. The C-reactive protein (PCR, mg/dL), Lactate dehydrogenase (LDH, U/L), White blood cell (WBC, 10^3^/mm^3^) count are routine laboratory tests. The P/F ratio represents the arterial oxygen pressure (PaO2 in mmHg) to fractional inspired oxygen (FiO2 expressed as a fraction, not a percentage). The results used in the rule, where the emergency department (ED) values not the peak values observed during the hospital stay. The ultrasound pattern was carried out in accordance with the use of lung ultrasound for COVID-19 patients proposed by Soldati G et al [19]. We defined “Wet/Interstitial syndrome” pattern when the operator highlighted B lines, pleura line broken and below the breaking point small to large consolidated areas (score 2 and 3) (Fig. 1); ‘‘Dry/Interstitial syndrom’’ pattern when the pleura line was continuous, regular or indented with visible vertical areas of white below the indent (B lines). B lines reflect local alterations in the acoustical properties of the lung caused by a replacement of air by water, blood, or fibrous tissue [8–10]. Besides, if the “Wet pattern” was localized to one segment of one lung, the whole ultrasound pattern in that patient was considered “Wet”.

### Laboratory confirmation

Laboratory confirmation of SARS-CoV-2 was performed at the Virology Laboratory of the University of Milan and at the Virology Laboratory of the San Matteo Hospital in Pavia, Ospedale Niguarda Ca’ Granda and Ospedale Maggiore Policlinico in Milan.RT-PCR assays were performed in accordance with the protocol established by the WHO [7].

### Baseline predictor variables and outcomes measures

To derive our prediction rule, we used clinical variables routinely available at presentation that were previously shown to be associated with mortality in patients with Covid-19 or other acute diseases [11]. These variables included demographics, comorbid conditions, physical examination findings, and laboratory findings. We extracted the clinical symptoms or signs, and laboratory findings on admission from electronic medical records and all laboratory testing was performed according to the clinical care needs of the patient. Laboratory assessments consisted of a complete blood count, blood chemical analysis, coagulation testing, assessment of liver and renal function, and measures of electrolytes, C-reactive protein and lactate dehydrogenase. The study outcomes used to derive our prediction rule was in-hospital mortality from all cause. We also assessed ICU admission and discharge.

### Statistical analysis

The study cohort consists of 119 consecutive patients with COVID-19 admitted to the same hospital. 79 consecutive patients (66%) were selected for the derivation of the model and 40 (34%) for its internal validation. The patients of the derivation cohort were in-patients of the Internal Medicine ward, those including into the validation cohort were mainly in-patients of other COVID-19 departments of the same Hospital. The variables that were previously described as predictors [11] and that were included in the prediction model have been selected by a wider list of variables trough the multiple linear regression. The constant and the ß-coefficients that the regression revealed were used to generate a score for each patient. A clustering of the scores divided patients into four specific groups (group I, group II, group III, group IV). To assess the discriminatory power that the model has to predict outcomes, the study presents a comparison between the ROC curve of each sample, with in-hospital mortality as the positive value. All the analyses have been performed trough Microsoft Excel 2016 and IBM SPSS Software.

## Results

### Characteristics of the patients included

79 patients with COVID-19 were included in derivation sample and 40 patients in the validation sample. Most patients were men (66%), with a mean age of 68 years (31–91) and 75 (62.5%) patients suffered from Arterial Hypertension (Table 1); 8 variables were associated with outcomes (Discharge, Admission in ICU, Exitus) by multiple regression analysis. Each weighted variable of the score system is illustrated in Table 2. The prediction rule classified similar proportions of patients in each of the four groups across the derivation and internal validation samples (Table 3).

**Table 1:** Baseline patient characteristics in the derivation and validation samples

Patients characteristics	Derivation samples (*n* = 79)	Validation samples (*n* = 40)
Age (min–max, mean)	31–91 (66.8)	44–93 (71)
Male sex (%)	54 (68%)	26 (65%)
Hypertension (%)	56 (70%)	19 (48%)
Temperature > 37.5 (%)	65 (82%)	31 (78%)
Pulmonary pattern “Wet” (%)	28 (35%)	18 (45%)
Respiratory rate (min–max, mean, median)	15–48 (26;24)	18–37 (24;24)
Arterial oxygen saturation (min–max, mean, median)	63–96 (88;88)	70–98 (90;91)
Absolute white blood cell count (10^3^/mm^3^) (min–max, mean, median)	1.1–16.1 (8.06;7.06)	3.21–21 (8,1;6,6)
CRP (mg/dL) (min–max, mean, median)	0.1–41 (12.8;11)	0.16–31.5 (12;10,5)
LDH (U/L) (min–max, mean, median)	87–1602 (504;420)	173–1340 (418;369)
BMI (kg/m^2^) (min–max, mean, median)	18.4–37 (26;26)	18–42 (27;26)
P/F Ratio (min–max, mean, median)	50–460 (243;252)	98–453 (272;284)
Lactates > 20 mg/dL (min–max, mean, median)	2.3–47.7 (13.78;11)	5–32 (13;11)

P/F ratio, the arterial oxygen pressure divided by the FIO_2_ (the fraction of inspired oxygen expressed as a decimal). *CRP* C-reactive protein, *LDH* lactate dehydrogenase, *BMI* body mass index

**Table 2:** Multivariable predictors of outcomes in the derivation cohort and their respective weights

Variables	B-coefficients	95%CI	*p* value
Fever for more than 5 days	0.219	− 0.15 to 0.59	0.24
Hypertension	0.194	− 0.12 10 0.51	0.22
Pattern US “Wet”	0.731	0.42–1.03	< 0.001
P/F ratio	0.002	0.00–0.003	0.02
Lactates (mg/dL)	0.041	0.02–0.06	< 0.001
WBC (G/L)	− 0.022	− 0.07 to 0.02	0.36
CRP (mg/dL)	0.019	0.00–0.03	0.02
Age	0.014	0.00–0.02	0.02

**Table 3:** Risk class distribution in the derivation and validation sample

Groups (score)	Range formula^a^	Range values	Derivation sample *n* (%)	Validation sample *n* (%)
Group 1	$\left(\mu -2\times Sd\right);(\mu -Sd)$	0.3087–0.9391	12 (15%)	5 (12.5%)
Group 2	$\left(\mu -Sd\right);(\mu )$	0.9392–1.5696	33 (42%)	16 (40%)
Group 3	$\left(\mu \right);(\mu +Sd)$	1.5697–2.2000	20 (25%)	14 (35%)
Group 4	$\left(\mu +Sd\right);(\mu +2\times Sd)$	2.2001–2.8304	14 (18%)	5 (12.5%)

^a^μ is the scores mean; SD is the scores standard deviation

### Determination of item weights and derivation of the Busto Score

The 8 patient variables independently associated with study outcomes included: fever for more than 5 days, Arterial Hypertension, Pulmonary ultrasound pattern (“wet” or “dry”), P/F index, Lactates, withe blood cells count (WBC), C-reactive protein (CRP) and Age. These clinical and laboratory variables were significantly associated with outcomes in the multiple model, and the R square of the model was 0.567 (Significance *F* < 0.001). The scoring system shown in Table 2 was used to quantify the magnitude of the association of each of these 8 factors with study outcomes. In the derivation cohort, Busto COVID-19 Score was correlated with “outcomes “, as expected (Pearson value = 0.75, *p* < 0,001). The procedure to get the score for each patient (${Score}_{patient A}$) is described by the following formula, where $\vartheta$ is a constant ( -0,998), ${V}_{n}$ is the value of each variable and ${\beta }_{n}$ is its relative $\beta$-coefficient:

$${\mathrm{Score}}_{p\mathrm{atient}\times A}=\vartheta +{V}_{1}\times {\beta }_{1}+ {V}_{2}\times {\beta }_{2}+\dots + {V}_{n}\times {\beta }_{n}$$

To get 4 different groups out of the scores that we obtained we decided to use the standard deviation method. We considered the mean as the threshold and we created the clusters as described in Table 3. To quickly calculate the score, you can connect to the website www.health-key.it.

### Internal validation of the prediction rule

**Fig. 2:**
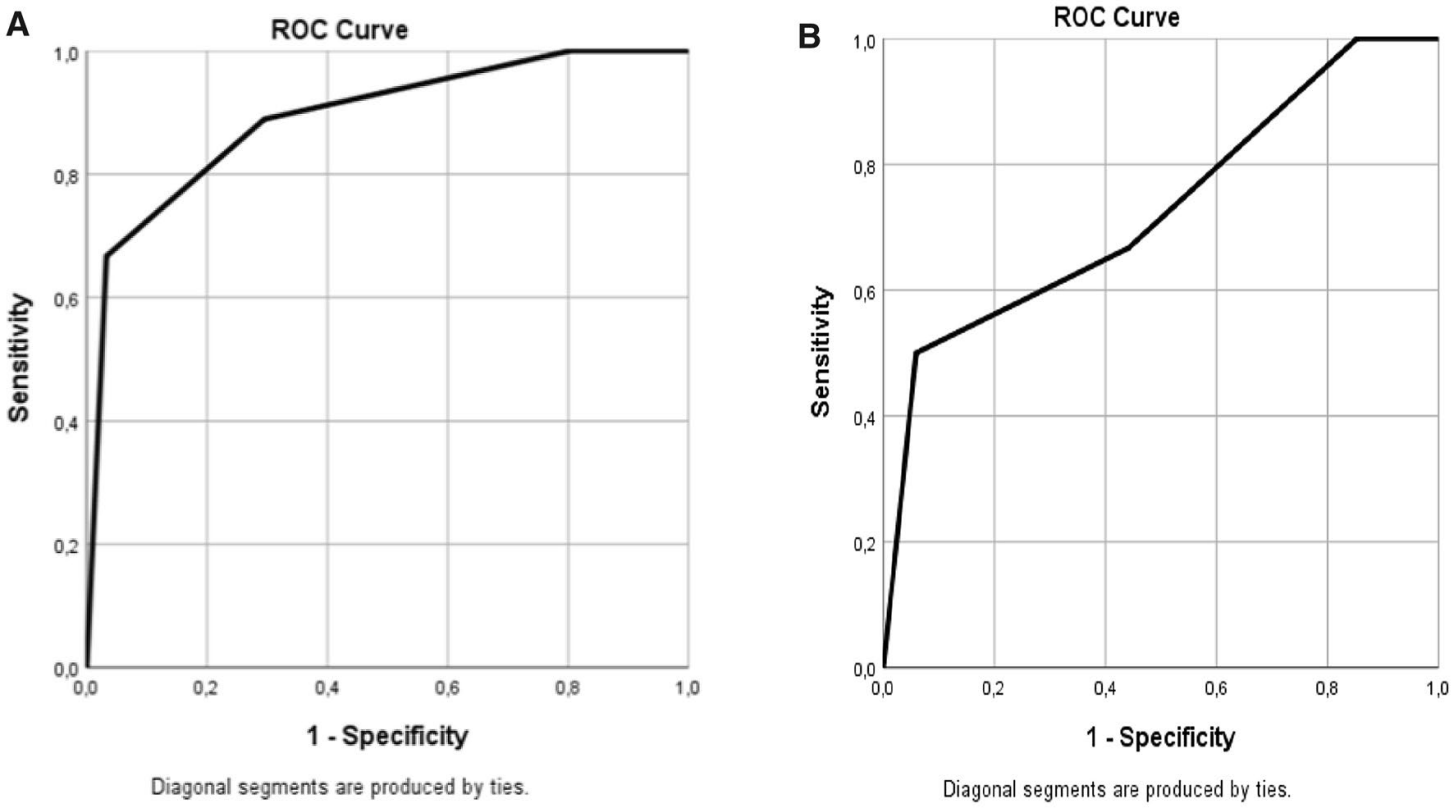
ROC curves of the derivation (A) and validation sample (B) about outcome “Exitus”

Once we got the prediction model trough the multivariable regression and the weighted combination of the variables, we run further analysis on the internal validation cohort. The prediction rule classified similar proportions of patients in each of the four groups across the derivation and internal validation samples (Table 4) and in-hospital mortality rate were 22% and 15%, respectively. Moreover, in each group was not significantly different between the derivation and the validation samples; in the internal validation sample, these rates ranged from 0% in the group 1 patients to 60% in the group 4 patients. The rule’s discriminatory power for mortality was similar in the derivation and internal validation samples, with an area under the receiver operating characteristic curve (ROC curve) of 0.90 (CI95% 0.801–0.982) and 0.73 (CI95% 0.484–0.972), respectively (Fig. 2).

**Table 4:** Risk class-specific medical outcomes in the derivation and validation samples

Medical outcomes	Derivation sample (*n* = 79)	Validation sample (*n* = 40)	*p* value
Discharged	52 (66% of the sample)	30 (75% of the sample)	0.85
Group 1	12 (100%)	5 (100%)	
Group 2	28 (84%)	11 (69%)	
Group 3	11 (55%)	12 (85%)	
Group 4	1(7%)	2 (40%)	
Admittend in ICU	9 (11% of the sample)	4 (10% of the sample)	0.42
Group 1	0%	0%	
Group 2	3 (9%)	3 (18.75%)	
Group 3	5 (25%)	1 (7%)	
Group 4	1 (7%)	0%	
Exitus	18 (22% of the sample)	6 (15% of the sample)	0.12
Group 1	0%	0%	
Group 2	2(6%)	2 (12.5%)	
Group 3	4(20%)	1 (7%)	
Group 4	12(86%)	3 (60%)

## Discussion

The COVID-19 outbreak put high pressure on Lombardy healthcare services. To prioritize resources for patients with the highest risk mortality, we developed a clinical prediction rule for prognosis of COVID-19 patients and a calculator to allow clinicians to calculate the likelihood (with 95% CI) that a hospitalized patient could develop critical illness. We identified 8 clinical and laboratory variables that stratify patients into 4 groups with increasing risk of death and other adverse medical outcomes (admission in ICU and mechanical ventilation). Yan L et al. proposed a prediction model to precisely and quickly quantify the risk of death only by laboratory values (LDH, hs-CRP, Lymphocites) [18]. Besides, Liang W et al. developed an online calculator to enter the values of 10 variables including X-ray pattern. Our specific experience allowed us to use, instead of the X-ray pattern, the easier disposable and user-friendly ultrasound tool. The performance of the rule, once validated in a retrospectively identified internal validation sample, was reliable. In the derivation and validation samples, we didn’t observe any significant difference between risk groups considering specific mortality, ICU admission and discharge. Our rule accurately identifies patients who are at low risk of fatal medical outcomes: group 1 and group 2 patients, respectively, had 0% and 6–12% or less in-hospital mortality. As the current pandemia imposed considerable resource limitations, our rule can provides clinicians with an explicit tool to identifying low-risk patients with COVID-19 who might be potential candidates for outpatient treatment or early hospital discharge. In this subgroup of patients, we observed an in-hospital mortality and an estimated ICU referral of 0%. Anyway, it’s mandatory to verify whether selected patients can be safely managed outside the ICU. However, group 4 patients had up to 86% in-hospital mortality rates and up to 25% ICU admission and mechanical ventilation rates. The intermediate groups (groups 2 and 3) are the most numerous and probably correspond to the overlap subset identified by the Siddiki model [17]. We believe that this is probably the point, where an adequate therapeutic approach can interrupt a process that leads to severe hyperinflammatory syndrome. Our prediction rule has several distinctive strengths: first, it consists of clearly defined and routinely available predictors; second, it concerns a wide spectrum of the disease, ranging from mild symptoms to severe acute respiratory failure with invasive mechanical ventilation. In conclusion, we suggest a practical tool for risk stratification that classifies patients with COVID-19 at increasing risk of death and other adverse outcomes. It can improve outpatient management and early hospital discharge of patients with COVID-19 identified as low risk (group 1 and group 2) with large cost savings without added risk. However, the dataset from a single hospital and the reduced number of cases represent two important limitations of this work. The preliminary results obtained from our experience require further external validation on a larger sample of patients.

## Conclusions

The prediction model that the study presents identifies COVID-19 patients with low risk of in-hospital mortality and admission to intensive care unit (ICU). Moreover, it establishes an intermediate portion of patients that should be treated accurately to avoid an unfavourable clinical evolution.
